# Supplementary material for: Dynalign II: common secondary structure prediction for RNA homologs with domain insertions
Source: Nucleic Acids Res. 2014 Nov 21;42(22):13939–48. doi: 10.1093/nar/gku1172 (PMC4267632; doi:10.1093/nar/gku1172)
Supplement: SUPPLEMENTARY DATA [file supp_gku1172_nar-02021-z-2014-File012.zip › manual/javadoc/RNAstructure_java_interface/source/menus/NucleicAcidMenu.html]

NucleicAcidMenu


JavaScript is disabled on your browser.


- Overview
- Package
- Class
- Tree
- Index
- Help

- Prev Class
- Next Class

- Frames
- No Frames

- All Classes

- Summary:
- Nested |
- Field |
- Constr |
- Method

- Detail:
- Field |
- Constr |
- Method


RNAstructure\_java\_interface.source.menus

## Class NucleicAcidMenu

- java.lang.Object
- - java.awt.Component
  - - java.awt.Container
    - - javax.swing.JComponent
      - - javax.swing.AbstractButton
        - - javax.swing.JMenuItem
          - - javax.swing.JMenu
            - - RNAstructure\_java\_utilities.RNAstructureMenu
              - - RNAstructure\_java\_interface.source.menus.RolloverMenu
                - - RNAstructure\_java\_interface.source.menus.NucleicAcidMenu

- All Implemented Interfaces:
  :   ActionListener, ImageObserver, ItemSelectable, MenuContainer, Serializable, EventListener, Accessible, MenuElement, SwingConstants

  ---

    

  ```
  public class NucleicAcidMenu
  extends RolloverMenu
  ```

  A class that creates a menu which holds commands specific for a nucleic
  acid type.
    
    
  Most File menus are identical, but items can be added to or subtracted from
  them in specific contexts.

  See Also:
  :   Serialized Form

- - ### Nested Class Summary

    - ### Nested classes/interfaces inherited from class RNAstructure\_java\_interface.source.menus.RolloverMenu

      `RolloverMenu.RolloverListener`
    - ### Nested classes/interfaces inherited from class javax.swing.JMenu

      `JMenu.AccessibleJMenu, JMenu.WinListener`
    - ### Nested classes/interfaces inherited from class javax.swing.JMenuItem

      `JMenuItem.AccessibleJMenuItem`
    - ### Nested classes/interfaces inherited from class javax.swing.AbstractButton

      `AbstractButton.AccessibleAbstractButton, AbstractButton.ButtonChangeListener`
    - ### Nested classes/interfaces inherited from class javax.swing.JComponent

      `JComponent.AccessibleJComponent`
    - ### Nested classes/interfaces inherited from class java.awt.Container

      `Container.AccessibleAWTContainer`
    - ### Nested classes/interfaces inherited from class java.awt.Component

      `Component.AccessibleAWTComponent, Component.BaselineResizeBehavior, Component.BltBufferStrategy, Component.FlipBufferStrategy`
  - ### Field Summary

    Fields

    | Modifier and Type | Field and Description |
    | --- | --- |
    | `private static long` | `serialVersionUID` |

    - ### Fields inherited from class javax.swing.JMenu

      `popupListener`
    - ### Fields inherited from class javax.swing.AbstractButton

      `actionListener, BORDER_PAINTED_CHANGED_PROPERTY, changeEvent, changeListener, CONTENT_AREA_FILLED_CHANGED_PROPERTY, DISABLED_ICON_CHANGED_PROPERTY, DISABLED_SELECTED_ICON_CHANGED_PROPERTY, FOCUS_PAINTED_CHANGED_PROPERTY, HORIZONTAL_ALIGNMENT_CHANGED_PROPERTY, HORIZONTAL_TEXT_POSITION_CHANGED_PROPERTY, ICON_CHANGED_PROPERTY, itemListener, MARGIN_CHANGED_PROPERTY, MNEMONIC_CHANGED_PROPERTY, model, MODEL_CHANGED_PROPERTY, PRESSED_ICON_CHANGED_PROPERTY, ROLLOVER_ENABLED_CHANGED_PROPERTY, ROLLOVER_ICON_CHANGED_PROPERTY, ROLLOVER_SELECTED_ICON_CHANGED_PROPERTY, SELECTED_ICON_CHANGED_PROPERTY, TEXT_CHANGED_PROPERTY, VERTICAL_ALIGNMENT_CHANGED_PROPERTY, VERTICAL_TEXT_POSITION_CHANGED_PROPERTY`
    - ### Fields inherited from class javax.swing.JComponent

      `accessibleContext, listenerList, TOOL_TIP_TEXT_KEY, ui, UNDEFINED_CONDITION, WHEN_ANCESTOR_OF_FOCUSED_COMPONENT, WHEN_FOCUSED, WHEN_IN_FOCUSED_WINDOW`
    - ### Fields inherited from class java.awt.Component

      `BOTTOM_ALIGNMENT, CENTER_ALIGNMENT, LEFT_ALIGNMENT, RIGHT_ALIGNMENT, TOP_ALIGNMENT`
    - ### Fields inherited from interface javax.swing.SwingConstants

      `BOTTOM, CENTER, EAST, HORIZONTAL, LEADING, LEFT, NEXT, NORTH, NORTH_EAST, NORTH_WEST, PREVIOUS, RIGHT, SOUTH, SOUTH_EAST, SOUTH_WEST, TOP, TRAILING, VERTICAL, WEST`
    - ### Fields inherited from interface java.awt.image.ImageObserver

      `ABORT, ALLBITS, ERROR, FRAMEBITS, HEIGHT, PROPERTIES, SOMEBITS, WIDTH`
  - ### Constructor Summary

    Constructors

    | Modifier | Constructor and Description |
    | --- | --- |
    | `private` | `NucleicAcidMenu(String acid)` Constructor. |
  - ### Method Summary

    Methods

    | Modifier and Type | Method and Description |
    | --- | --- |
    | `static NucleicAcidMenu` | `createDNA()` Create a DNA menu. |
    | `static NucleicAcidMenu` | `createRNA()` Create a RNA menu. |
    | `protected void` | `doMenuActions(String command)` Do any actions specific to this menu. |

    - ### Methods inherited from class RNAstructure\_java\_interface.source.menus.RolloverMenu

      `convertToolTips`
    - ### Methods inherited from class RNAstructure\_java\_utilities.RNAstructureMenu

      `actionPerformed, addCheckBoxMenuItem, addMenuItem, addMenuItem, addMenuItem, insertMenuItem`
    - ### Methods inherited from class javax.swing.JMenu

      `add, add, add, add, add, addMenuListener, addSeparator, applyComponentOrientation, createActionChangeListener, createActionComponent, createWinListener, doClick, fireMenuCanceled, fireMenuDeselected, fireMenuSelected, getAccessibleContext, getComponent, getDelay, getItem, getItemCount, getMenuComponent, getMenuComponentCount, getMenuComponents, getMenuListeners, getPopupMenu, getPopupMenuOrigin, getSubElements, getUIClassID, insert, insert, insert, insertSeparator, isMenuComponent, isPopupMenuVisible, isSelected, isTearOff, isTopLevelMenu, menuSelectionChanged, paramString, processKeyEvent, remove, remove, remove, removeAll, removeMenuListener, setAccelerator, setComponentOrientation, setDelay, setMenuLocation, setModel, setPopupMenuVisible, setSelected, updateUI`
    - ### Methods inherited from class javax.swing.JMenuItem

      `actionPropertyChanged, addMenuDragMouseListener, addMenuKeyListener, configurePropertiesFromAction, fireMenuDragMouseDragged, fireMenuDragMouseEntered, fireMenuDragMouseExited, fireMenuDragMouseReleased, fireMenuKeyPressed, fireMenuKeyReleased, fireMenuKeyTyped, getAccelerator, getMenuDragMouseListeners, getMenuKeyListeners, init, isArmed, processKeyEvent, processMenuDragMouseEvent, processMenuKeyEvent, processMouseEvent, removeMenuDragMouseListener, removeMenuKeyListener, setArmed, setEnabled, setUI`
    - ### Methods inherited from class javax.swing.AbstractButton

      `addActionListener, addChangeListener, addImpl, addItemListener, checkHorizontalKey, checkVerticalKey, createActionListener, createActionPropertyChangeListener, createChangeListener, createItemListener, doClick, fireActionPerformed, fireItemStateChanged, fireStateChanged, getAction, getActionCommand, getActionListeners, getChangeListeners, getDisabledIcon, getDisabledSelectedIcon, getDisplayedMnemonicIndex, getHideActionText, getHorizontalAlignment, getHorizontalTextPosition, getIcon, getIconTextGap, getItemListeners, getLabel, getMargin, getMnemonic, getModel, getMultiClickThreshhold, getPressedIcon, getRolloverIcon, getRolloverSelectedIcon, getSelectedIcon, getSelectedObjects, getText, getUI, getVerticalAlignment, getVerticalTextPosition, imageUpdate, isBorderPainted, isContentAreaFilled, isFocusPainted, isRolloverEnabled, paintBorder, removeActionListener, removeChangeListener, removeItemListener, removeNotify, setAction, setActionCommand, setBorderPainted, setContentAreaFilled, setDisabledIcon, setDisabledSelectedIcon, setDisplayedMnemonicIndex, setFocusPainted, setHideActionText, setHorizontalAlignment, setHorizontalTextPosition, setIcon, setIconTextGap, setLabel, setLayout, setMargin, setMnemonic, setMnemonic, setMultiClickThreshhold, setPressedIcon, setRolloverEnabled, setRolloverIcon, setRolloverSelectedIcon, setSelectedIcon, setText, setUI, setVerticalAlignment, setVerticalTextPosition`
    - ### Methods inherited from class javax.swing.JComponent

      `addAncestorListener, addNotify, addVetoableChangeListener, computeVisibleRect, contains, createToolTip, disable, enable, firePropertyChange, firePropertyChange, firePropertyChange, fireVetoableChange, getActionForKeyStroke, getActionMap, getAlignmentX, getAlignmentY, getAncestorListeners, getAutoscrolls, getBaseline, getBaselineResizeBehavior, getBorder, getBounds, getClientProperty, getComponentGraphics, getComponentPopupMenu, getConditionForKeyStroke, getDebugGraphicsOptions, getDefaultLocale, getFontMetrics, getGraphics, getHeight, getInheritsPopupMenu, getInputMap, getInputMap, getInputVerifier, getInsets, getInsets, getListeners, getLocation, getMaximumSize, getMinimumSize, getNextFocusableComponent, getPopupLocation, getPreferredSize, getRegisteredKeyStrokes, getRootPane, getSize, getToolTipLocation, getToolTipText, getToolTipText, getTopLevelAncestor, getTransferHandler, getVerifyInputWhenFocusTarget, getVetoableChangeListeners, getVisibleRect, getWidth, getX, getY, grabFocus, isDoubleBuffered, isLightweightComponent, isManagingFocus, isOpaque, isOptimizedDrawingEnabled, isPaintingForPrint, isPaintingOrigin, isPaintingTile, isRequestFocusEnabled, isValidateRoot, paint, paintChildren, paintComponent, paintImmediately, paintImmediately, print, printAll, printBorder, printChildren, printComponent, processComponentKeyEvent, processKeyBinding, processMouseEvent, processMouseMotionEvent, putClientProperty, registerKeyboardAction, registerKeyboardAction, removeAncestorListener, removeVetoableChangeListener, repaint, repaint, requestDefaultFocus, requestFocus, requestFocus, requestFocusInWindow, requestFocusInWindow, resetKeyboardActions, reshape, revalidate, scrollRectToVisible, setActionMap, setAlignmentX, setAlignmentY, setAutoscrolls, setBackground, setBorder, setComponentPopupMenu, setDebugGraphicsOptions, setDefaultLocale, setDoubleBuffered, setFocusTraversalKeys, setFont, setForeground, setInheritsPopupMenu, setInputMap, setInputVerifier, setMaximumSize, setMinimumSize, setNextFocusableComponent, setOpaque, setPreferredSize, setRequestFocusEnabled, setToolTipText, setTransferHandler, setUI, setVerifyInputWhenFocusTarget, setVisible, unregisterKeyboardAction, update`
    - ### Methods inherited from class java.awt.Container

      `add, add, add, addContainerListener, addPropertyChangeListener, addPropertyChangeListener, areFocusTraversalKeysSet, countComponents, deliverEvent, doLayout, findComponentAt, findComponentAt, getComponent, getComponentAt, getComponentAt, getComponentCount, getComponents, getComponentZOrder, getContainerListeners, getFocusTraversalKeys, getFocusTraversalPolicy, getLayout, getMousePosition, insets, invalidate, isAncestorOf, isFocusCycleRoot, isFocusCycleRoot, isFocusTraversalPolicyProvider, isFocusTraversalPolicySet, layout, list, list, locate, minimumSize, paintComponents, preferredSize, printComponents, processContainerEvent, processEvent, removeContainerListener, setComponentZOrder, setFocusCycleRoot, setFocusTraversalPolicy, setFocusTraversalPolicyProvider, transferFocusDownCycle, validate, validateTree`
    - ### Methods inherited from class java.awt.Component

      `action, add, addComponentListener, addFocusListener, addHierarchyBoundsListener, addHierarchyListener, addInputMethodListener, addKeyListener, addMouseListener, addMouseMotionListener, addMouseWheelListener, bounds, checkImage, checkImage, coalesceEvents, contains, createImage, createImage, createVolatileImage, createVolatileImage, disableEvents, dispatchEvent, enable, enableEvents, enableInputMethods, firePropertyChange, firePropertyChange, firePropertyChange, firePropertyChange, firePropertyChange, firePropertyChange, getBackground, getBounds, getColorModel, getComponentListeners, getComponentOrientation, getCursor, getDropTarget, getFocusCycleRootAncestor, getFocusListeners, getFocusTraversalKeysEnabled, getFont, getForeground, getGraphicsConfiguration, getHierarchyBoundsListeners, getHierarchyListeners, getIgnoreRepaint, getInputContext, getInputMethodListeners, getInputMethodRequests, getKeyListeners, getLocale, getLocation, getLocationOnScreen, getMouseListeners, getMouseMotionListeners, getMousePosition, getMouseWheelListeners, getName, getParent, getPeer, getPropertyChangeListeners, getPropertyChangeListeners, getSize, getToolkit, getTreeLock, gotFocus, handleEvent, hasFocus, hide, inside, isBackgroundSet, isCursorSet, isDisplayable, isEnabled, isFocusable, isFocusOwner, isFocusTraversable, isFontSet, isForegroundSet, isLightweight, isMaximumSizeSet, isMinimumSizeSet, isPreferredSizeSet, isShowing, isValid, isVisible, keyDown, keyUp, list, list, list, location, lostFocus, mouseDown, mouseDrag, mouseEnter, mouseExit, mouseMove, mouseUp, move, nextFocus, paintAll, postEvent, prepareImage, prepareImage, processComponentEvent, processFocusEvent, processHierarchyBoundsEvent, processHierarchyEvent, processInputMethodEvent, processMouseWheelEvent, remove, removeComponentListener, removeFocusListener, removeHierarchyBoundsListener, removeHierarchyListener, removeInputMethodListener, removeKeyListener, removeMouseListener, removeMouseMotionListener, removeMouseWheelListener, removePropertyChangeListener, removePropertyChangeListener, repaint, repaint, repaint, resize, resize, setBounds, setBounds, setCursor, setDropTarget, setFocusable, setFocusTraversalKeysEnabled, setIgnoreRepaint, setLocale, setLocation, setLocation, setName, setSize, setSize, show, show, size, toString, transferFocus, transferFocusBackward, transferFocusUpCycle`
    - ### Methods inherited from class java.lang.Object

      `clone, equals, finalize, getClass, hashCode, notify, notifyAll, wait, wait, wait`
    - ### Methods inherited from interface javax.swing.MenuElement

      `processKeyEvent, processMouseEvent`

- - ### Field Detail


    - #### serialVersionUID

      ```
      private static final long serialVersionUID
      ```

      See Also:
      :   Constant Field Values
  - ### Constructor Detail


    - #### NucleicAcidMenu

      ```
      private NucleicAcidMenu(String acid)
      ```

      Constructor.

      Parameters:
      :   `acid` - The nucleic acid type.
  - ### Method Detail


    - #### createDNA

      ```
      public static NucleicAcidMenu createDNA()
      ```

      Create a DNA menu.

      Returns:
      :   The DNA menu.


    - #### createRNA

      ```
      public static NucleicAcidMenu createRNA()
      ```

      Create a RNA menu.

      Returns:
      :   The RNA menu.


    - #### doMenuActions

      ```
      protected void doMenuActions(String command)
      ```

      **Description copied from class: `RNAstructureMenu`**

      Do any actions specific to this menu.

      **Overrides:**
      :   `doMenuActions` in class `RolloverMenu`

      Parameters:
      :   `command` - The command that signifies a particular action.


- Overview
- Package
- Class
- Tree
- Index
- Help

- Prev Class
- Next Class

- Frames
- No Frames

- All Classes

- Summary:
- Nested |
- Field |
- Constr |
- Method

- Detail:
- Field |
- Constr |
- Method
